# Effects of an open-label pilot study with high-dose EPA/DHA concentrates on plasma phospholipids and behavior in children with attention deficit hyperactivity disorder

**DOI:** 10.1186/1475-2891-6-16

**Published:** 2007-07-13

**Authors:** Paul J Sorgi, Edward M Hallowell, Heather L Hutchins, Barry Sears

**Affiliations:** 1 Hallowell Center, 142 North Road, Suite F 105, Sudbury, MA 01776, USA; 2 Inflammation Research Foundation, 222 Rosewood Drive, Suite 500, Danvers, MA 01923, USA

## Abstract

**Background:**

Attention deficit hyperactivity disorder (ADHD) is the most common neurological condition in children. This pilot study evaluated the effects of high-dose eicosapentaenoic acid (EPA) and docosahexaenoic acid (DHA) supplementation on the isolated plasma phospholipids and behavior in children with ADHD (primarily inattentive subtype and combined subtype).

**Methods:**

Nine children were initially supplemented with 16.2 g EPA/DHA concentrates per day. The dosage was adjusted dependent on the ratio of arachidonic acid (AA) to EPA in the isolated plasma phospholipids at four weeks to reach a level normally found in the Japanese population.

**Results:**

At the end of the eight-week study, supplementation resulted in significant increases in EPA and DHA, as well as a significant reduction in the AA:EPA ratio (20.78 ± 5.26 to 5.95 ± 7.35, p < 0.01). A psychiatrist (blind to supplement compliance or dosage modifications) reported significant improvements in behavior (inattention, hyperactivity, oppositional/defiant behavior, and conduct disorder). There was also a significant correlation between the reduction in the AA:EPA ratio and global severity of illness scores.

**Conclusion:**

The findings of this small pilot study suggest supplementation with high-dose EPA/DHA concentrates may improve behavior in children with ADHD.

## Background

Attention deficit hyperactivity disorder (ADHD) is a neurological condition characterized by the inability to concentrate in a sustained manner, to pay attention to tasks, and to control impulsive actions [1]. It is estimated that 3 to 7 percent of children have this disorder, and boys are affected to a much greater extent than girls [2]. As many as 60 to 80 percent of children with ADHD continue to have problems with this condition as they become adults [1]. The etiology appears to be multi-factorial with both genetic and environmental influences. Among these influences is an observed decrease in long-chain (LC) polyunsaturated fatty acids (PUFAs) in children with ADHD. Some proposed mechanisms for the low levels of PUFAs include insufficient dietary intake, inefficient conversion of shorter chain PUFAs to LC PUFAs or rapid metabolism of LC PUFAs [3]. Stevens et al. [4,5] found that young boys with ADHD and symptoms of essential fatty acid (EFA) deficiency (excessive thirst, dry skin and hair, brittle nails, frequent urination and/or hyperfollicular keratoses) are characterized by low levels of LC PUFAs, including AA, EPA and DHA in the plasma phospholipids compared to control. This group of children with ADHD also had a high ratio of AA to EPA compared to control (68.87 vs. 45.83 respectively) suggesting the depression of EPA was greater than that of AA [4,5]. Other studies from the same group have also reported greater AA:EPA ratios in children [3] and young adults [6] with ADHD compared to control.

The findings that children with ADHD have altered PUFA levels led to interventional studies that supplemented with these fatty acids. Hirayama et al. [7] found that supplementation of 0.61 g of LC omega-3 fatty acids per day for two months (primarily DHA in foods) had no effect when analyzing parent and teacher assessments separately in children with ADHD; however, when analyzing assessments together, physical aggression significantly improved compared to the placebo group [8]. Voigt et al. [9] supplemented children with ADHD for four months with 0.35 g of DHA per day and found no improvements in any ADHD symptoms. Richardson and Puri [10] supplemented children with ADHD related symptoms, primarily dyslexia, for three months with 1.67 g per day of omega-3 and omega-6 fatty acids or olive oil placebo in a double-blind randomized fashion. Half of the scales tested (seven of 14) improved compared to placebo, including cognition, anxiety/shyness, psychosomatic subscales, restlessness/impulsivity and three global scales; however, the participants were children with ADHD-related symptoms not diagnosed with ADHD in accordance to DSM IV criteria. The only study that measured the AA:EPA ratio was that of Stevens et al. [11] who supplemented children with ADHD and symptoms of EFA deficiency with 0.66 g per day of both omega-3 and omega-6 PUFAs for four months. Although the AA:EPA ratio decreased from 33.04 to 15.19, only two of 16 behavioral outcome measures significantly improved compared to the placebo group suggesting the decreased AA:EPA ratio may not have been lowered enough to observe a greater impact on behavior.

Depression is often a co-morbidity of ADHD and an increased AA:EPA ratio has been shown to positively correlate with severity of depression [12]. High-dose dietary supplementation with EPA and DHA (9.6 g) has been shown as an effective adjunctive treatment for bipolar depression [13]. Epidemiological data has shown that the Japanese population has low rates of depression [14], compared to the US population and they have a high intake of fish and low AA:EPA ratio [15]. The ratio of AA:EPA in the isolated plasma phospholipids of the Japanese population is approximately 1.3 to 3 [15,16]; young boys with ADHD in the United States had AA:EPA ratios greater than 30 [4,5,11]. A recent study found negative correlations between omega-3 status and behavior in young adults with ADHD suggesting lower omega-3 status may be associated with severity of behavioral symptoms [6].

The interventional studies in children with ADHD demonstrate that some behavior may improve with PUFA supplementation [5,8,10]; however, the findings have not been consistent and few have monitored fatty acid levels in the plasma phospholipids. A possible association between ADHD behaviors and omega-3 status, particularly the AA:EPA ratio and the lack of consistent results led us to hypothesize that insufficient levels of omega-3 fatty acids, or lack of sufficient reduction of the AA:EPA ratio, are two possible explanations for the inconsistent findings of previous studies. To address this hypothesis, we undertook an open-label pilot study to first determine if children with ADHD would adhere to a protocol of high-dose EPA/DHA concentrates (initial dosage of 16.2 g EPA and DHA per day) to reach the goal AA:EPA ratio between 1.5 and 3 as found in the Japanese population. Second, behavior was assessed by a psychiatrist specialized in this childhood disorder to measure what effect such a reduced AA:EPA ratio would have on behavior.

## Methods

### Study design and participants

This was an eight-week, open-label, proof-of-efficacy pilot study. Nine children aged 8–16 were recruited from the patient population under treatment for ADHD-primarily inattentive subtype or ADHD-combined subtype at the Hallowell Center, Sudbury, MA. There were more boys (n = 6) than girls (n = 3). Two-thirds (n = 6) presented with ADHD-combined subtype, and one-third (n = 3) with ADHD-primarily inattentive subtype according to criteria of the Diagnostic Statistical Manual (DSM) IV [17]. Two of the three participants who presented with ADHD-primarily inattentive subtype were girls. Participant characteristics are outlined in Table 1. All participants had an established relationship with the psychiatrist involved in the study. Three participants voluntarily discontinued stimulant medication prior to study initiation with the psychiatrist's approval. The remainder continued with their treatment regime for the duration of the study. There were no medication dosage changes during the course of the eight-week study. Children and parents/guardians provided informed and written assent and consent respectively. Integreview, Houston, TX, approved the study for the use of human subjects in research.

**Table 1:** Baseline Characteristics of Study Participants*

Age (y)	11.44 ± 1.51
Gender (% male)	67
Race (% white)	100
School grade	6.00 ± 1.77
ADD %	33
ADHD %	67
Years since diagnosis	2.63 ± 1.41
Height (in)	61.67 ± 5.13
Weight (lbs)	113.67 ± 34.04
Taking stimulant	
medication (%)	67

* Reported values are means ± SD (n = 9) or percentages.

### Study intervention

At the start of the study, all participants were instructed to consume two tablespoons (30 mL) of a liquid EPA/DHA concentrate (supplied by the Inflammation Research Foundation) providing 16.2 g of LC omega-3 fatty acids (10.8 g EPA and 5.4 g DHA) per day. The EPA/DHA concentrate dosage was adjusted at week four based on the AA:EPA ratio in the isolated plasma phospholipids as follows: if the AA:EPA ratio was below 1.0, the dosage was decreased to 15 ml (5.4 g EPA, 2.7 g DHA per day), if the AA:EPA ratio was between 1.0 and 1.5 the dosage was decreased to 20 ml (8.1 g EPA, 4 g DHA per day). Isolated plasma phospholipids are not subject to daily fluctuations in dietary intake and are a reliable marker of fatty acid levels [18] and correlate to dietary intake of fatty fish [19-21] and fish oil supplementation [22,23]. EPA levels were used to monitor adherence to the supplementation protocol and the participant's parent/guardian was phoned once per week to monitor adherence and adverse effects of the EPA/DHA concentrates. The children and at least one parent/guardian met with the psychiatrist at three time points: baseline (week 0), midpoint (week four) and conclusion (week eight). At the initial (baseline) visit participants were advised to follow a "healthy diet" that encouraged fruits, vegetables and balanced intake of macronutrients at meals and snacks. At each of the three meetings, the psychiatrist conducted behavioral assessments, and a phlebotomist drew blood for fatty acid analysis. Fatty acid analysis of the isolated plasma phospholipids was completed by Nutrasource Diagnostics, Guelph, ON, Canada, as described by Laidlaw and Holub [23].

### Behavioral assessment

The ADHD Symptom Checklist-4 (ADHD SC-4) was used to monitor behavioral changes by the psychiatrist at each meeting. Retrospectively the psychiatrist asked the parent/guardian and child each of the checklist questions and also observed symptoms during the process. This questionnaire categorizes behavior as inattention, hyperactivity, oppositional/defiant, and conduct disorder. There was also a section to monitor medication side effects. Inattention and hyperactivity scores can range from 0 to 27 each. Oppositional/defiant scores range from 0 to 24 and conduct disorder from 0 to 3 [24,25].

The Clinical Global Impression Scale was used by the psychiatrist to rate participants' severity of illness [26]. The scores are derived from a 7-point Likert scale [26]. Severity of illness ranged from 1 as normal (not at all symptomatic), to 7 as among the most symptomatic patients. Although the psychiatrist knew the children were part of a study, he was blind to dosage adjustments and protocol adherence. The parents also completed the short form Conner's Parent's Rating Scale (CPRS) [27-29] at baseline, week four and week eight. Responses were scored and categorized into 4 groups: oppositional behavior, cognitive problems/inattention, hyperactivity and an ADHD index.

### Statistical analysis

Summary statistics are reported as mean ± SD and medians. Statistical analyses were performed using Stata for Mac (version 9, StataCorp LP, College Station, Texas). Fatty acids, ADHD SC-4, CPRS, and severity of illness were reported for baseline (week 0), midpoint (week four) and at the conclusion of the study (week eight). The non-parametric Friedman test was used to assess changes over time. If the Friedman test was statistically significant at the 0.05 level, then the Wilcoxon signed rank test was used as a post-hoc test to compare changes from baseline to four and eight weeks. Spearman correlations were used to identify a relationship between changes in the primary fatty acid outcome variable, the AA:EPA ratio, and the primary behavioral outcome variable, severity of illness.

## Results

### Effect of EPA/DHA concentrates on isolated plasma phospholipid fatty acid levels

**Figure 1:**
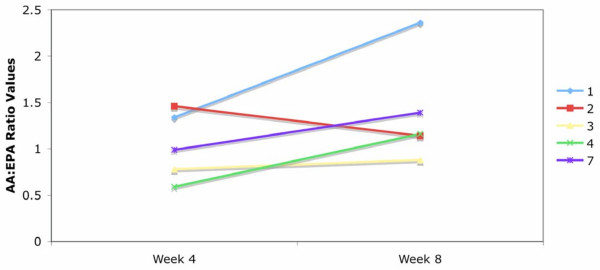
AA:EPA ratio changes from week 4 to 8 in the participants with EPA/DHA concentrate dosage changes.* * Each line represents a participant's AA:EPA ratio from week 4 to week 8 in the five who were instructed to decrease their intake of the EPA/DHA concentrate.

Blood was monitored throughout the study to ensure that the AA:EPA ratio was greater than 1.0 because of the potential concern with high-dose EPA/DHA supplementation on prolonged bleeding times; although, a recent study of the Japanese indicated no adverse effects related to EPA intake in patients whose AA:EPA ration was lowered to 0.8 [15]. To maintain the goal AA:EPA ratio between 1.5 and 3 dosage adjustments were made at week four based on fatty acid results. At week four, three participants had an AA:EPA ratio below 1.0 and adjusted their EPA/DHA concentrate dosage to 15 ml per day; two participants had AA:EPA ratios between 1.0 and 1.5 and adjusted their dosage to 20 ml EPA/DHA concentrate; the remaining four participants had an AA:EPA ratio of 1.5 or above, and were instructed to continue with the initial daily dosage. Four of the five participants whose dosage was adjusted at week four increased their AA:EPA ratio by week eight, however the AA:EPA ratio remained less than 3 (Figure 1). Two participants had an AA:EPA ratio less than one at week eight and upon exiting the study were advised to decrease the supplement dosage if they were to continue with the protocol post study.

Overall, at the conclusion of the eight weeks of supplementation, the average EPA and DHA levels in the isolated plasma phospholipids significantly increased by a factor of 9.5 and 2.4 respectively (Table 2). The AA tended to decrease at eight weeks, although the reduction was not significant (p = 0.07). As a consequence of AA and EPA changes, there was a 71% reduction in the mean AA:EPA ratio (20.78 ± 5.26 to 5.95 ± 7.35, p < 0.01) from baseline to week eight. The EPA/DHA concentrate dosage adjustment at week four resulted in the average EPA and DHA levels to increase from week four to eight with a corresponding 42% relative increase (4.19 ± .5.45 to 5.95 ± 7.35, p = 0.07) in the AA:EPA ratio; in contrast, the relative increase in median AA:EPA ratio over the same period was only 16% and better reflects the actual changes among compliant study participants. Nonetheless, these increases demonstrate sensitivity to EPA and DHA supplementation dosage.

**Table 2:** Fatty acids from the isolated plasma phospholipids described as means ± SD and median.*

Plasma FA		Baseline	Week 4	Week 8
	*n*-6			
18:2 (LA)		22.61 ± 1.39	17.81 ± 2.85	21.88 ± 4.89
median		21.95	17.49	21.08
20:3 (DGLA)		3.12 ± 0.44	1.86 ± 0.82	2.15 ± 0.64
median		3.09	2.25	2.05
20:4 (AA)		9.52 ± 0.70	8.92 ± 0.83	8.69 ± 1.30
median		9.34	8.92	8.37
	*n*-3			
18:3 (LNA)		0.13 ± 0.07	0.13 ± 0.07	0.13 ± 0.06
median		0.13	0.1	0.12
20:5 (EPA)		0.49 ± 0.12	5.89 ± 4.27^a^	4.64 ± 3.65^a^
median		0.46	6.13	4.72
22:6 (DHA)		2.30 ± 0.88	5.68 ± 1.28^a^	5.61 ± 2.14^a^
median		2.07	6.11	6.19
*Totals and ratios*				
Saturated		44.29 ± 0.99	43.94 ± 1.26	42.19 ± 1.05
median		44.2	44.04	42.53
Monounsaturated		15.43 ± 1.38	13.64 ± 1.18	12.31 ± 2.12
median		15.7	13.51	11.93
Polyunsaturated		40.27 ± 1.03	42.41 ± 1.43	45.51 ± 1.26
median		40.21	42.02	45.38
total *n*-6		36.37 ± 0.99	29.07 ± 4.31^a^	33.42 ± 5.67
median		36.57	29.54	31.59
total *n*-3		3.90 ± 0.87	13.35 ± 5.27^a^	12.09 ± 6.17^a^
median		3.66	12.74	13.38
*n*-6: *n*-3 ratio		9.74 ± 2.14	2.79 ± 1.88^a^	3.93 ± 2.73^a^
median		9.61	2.39	2.36
AA:EPA ratio		20.78 ± 5.26	4.19 ± 5.45^a^	5.95 ± 7.35^a^
median		20.14	1.46	1.69
AA:EPA ratio (n = 7)^#^		20.73 ± 5.26	2.53 ± 3.49^a^	2.52 ± 2.91
median		19.58	1.34	1.39

* Values are mean ± SD and medians (n = 9). ^a ^p < 0.01 using Wilcoxon signed rank test to compare to baseline. LA, linoleic acid; DGLA, dihomogammalinolenic acid; AA, arachidonic acid; LNA, linolenic acid; EPA, eicosapentaenoic acid; DHA, docosahexaenoic acid.

### Protocol adherence

**Figure 2:**
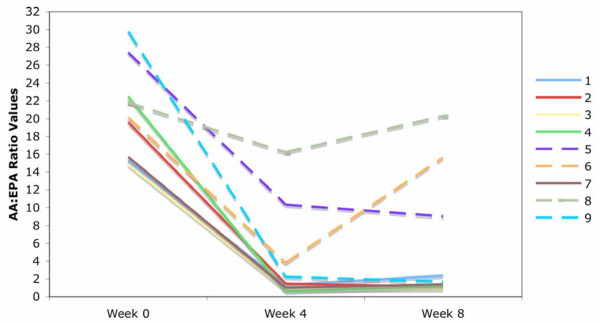
AA:EPA ratio changes at week 0, week 4 and week 8.* *Each line represents a participant's AA:EPA ratio from baseline to week 4 and week 8, dashed lines represent the participants whose EPA/DHA concentrate dosage was not changed at week 4.

The largest AA:EPA reduction for most participants occurred in the first four weeks (Figure 2). Figure 2 shows that one participant's AA:EPA ratio returned to baseline levels at week eight following a reduction at week four. Furthermore, this participant's EPA and DHA levels also returned to near baseline levels, indicating poor compliance from week four to eight. This participant's parent reported poor protocol adherence for the second half of the study, which mimics the fatty acid data. A second participant who refused to consume the liquid was switched to a capsule supplementation and was instructed to consume 24 one-gram capsules (9.6 g EPA and 4.8 g DHA) per day from week two to week eight. The lack of change in the AA:EPA ratio in this participant indicated non-compliance. We chose less than 100% increase in the isolated plasma phospholipids for EPA and DHA as an indicator of poor compliance. The two non-compliant children were not of the same ADHD subtype or gender. All other participants had greater than a 100% increase in both EPA and DHA levels at the conclusion of the study and were considered compliant with study supplementation protocol.

### Adverse effects

One participant reported loose stools while taking 30 ml of the liquid EPA/DHA concentrate per day. At week four, the dosage was decreased to 15 ml per day with no subsequent adverse events. No observational effects on bleeding were reported by the parents. One child had adverse effects (tics) related to stimulant medication prior to study initiation that continued throughout the study. Sleep disturbance is another know adverse effect of medication for the treatment for ADHD [30]; four of the nine children had improvements in sleep noted by the parents.

### Behavioral analysis

All categories of the ADHD SC-4 significantly improved by week eight. Specifically, inattention, hyperactivity, oppositional/defiant behavior and conduct disorder all significantly improved over the course of the study (Table 3). The CPRS showed significant improvements (p < 0.05) in all four categories (oppositional behavior, cognitive problems/inattention, hyperactivity, and the ADHD index) over the eight week study as well (Table 3). The severity of illness score, assessed by the psychiatrist, tended to improve (p = 0.08) over time. On average, the severity of illness score decreased by 1 point from 4.4 (moderately symptomatic) to 3.3 (mildly symptomatic). The two subjects who were identified as non-compliant from parental reports and fatty acid levels were the only participants who were scored a 5 (markedly symptomatic) at baseline and week eight.

**Table 3:** Behavioral assessment*

**Assessment**	**Baseline**	**Week 4**	**Week 8**
ADHD SC-4 Inattention	18.11 ± 5.37	10.56 ± 5.46^a^	9.78 ± 6.91^a^
median	17	9	10
ADHD SC-4 Hyperactivity	11.33 ± 4.53	7.67 ± 5.96	5.22 ± 3.99^a^
median	10	6	4
ADHD SC-4 Oppositional/defiant	10.11 ± 6.79	5.44 ± 3.88^b^	5.22 ± 3.56^b^
median	11	7	5
ADHD SC-4 Conduct disorder	5.22 ± 6.26	0.89 ± 0.93^b^	1.11 ± 2.26^b^
median	4	1	0
CPRS-Oppositional behavior	8.67 ± 4.58	5.38 ± 3.11	4.89 ± 2.93^a^
median	7	5.5	6
CPRS- Cognitive problems/inattention	11.56 ± 5.13	7.00 ± 3.89^b^	8.44 ± 6.48
median	12	8.5	8
CPRS- Hyperactivity	5.89 ± 3.79	2.63 ± 2.39	3.22 ± 4.49
median	6	3	2
CPRS- ADHD index	22.67 ± 9.03	13.63 ± 6.37^a^	15.44 ± 9.49^b^
median	22	13	14
Clinical Severity of Illness	4.38 ± 0.74	3.56 ± 0.73	3.33 ± 1.12^c^
median	4.5	3	3

* Values are mean ± SD and medians (n = 9). ADHD SC-4, Attention Deficit Hyperactivity Disorder Symptom Checklist 4. CPRS, Conners' Parents Rating Scale ^a ^p < 0.01, ^b ^p < 0.05 using Wilcoxon signed rank test to compare to baseline; ^c ^p = 0.08 using Friedman test to compare to baseline.

### Correlations

There was a significant positive correlation of the percent change in AA:EPA ratio with the percent change in severity of illness (Rho = 0.7638, p = 0.027). However, the AA:EPA ratio did not significantly correlate with the ADHD SC-4 or CPRS categories.

## Discussion

Supplementation with high-dose EPA/DHA concentrate resulted in significant modifications of fatty acids, particularly a significant improvement in the AA:EPA ratio in the isolated plasma phospholipids and improvements in behavior assessed by a psychiatrist (blinded to protocol adherence and supplement dosage adjustments) in this small pilot sample of children with ADHD.

At baseline fatty acid analysis of the isolated plasma phospholipids from the children in this study were similar to that of previous studies of children with ADHD and thirst/skin symptoms of EFA deficiency [3,5,11]; however, we did not assess EFA deficiency symptoms. Children with ADHD and thirst/skin symptoms of EFA deficiency had lower AA and DHA levels in the plasma phospholipids compared to control groups. Both the AA and DHA mean levels from previous studies [3,5,11] were within the 95% CI (8.98–10.05; 1.63–2.97, respectively) of this study's mean AA and DHA levels.

Supplementation of high-dose EPA/DHA concentrates resulted in marked changes in fatty acid levels of the isolated plasma phospholipids. EPA and DHA levels in the isolated plasma phospholipids were used to monitor compliance. We chose the AA:EPA ratio as an important marker because of it's relationship with depression [12], as depression is often associated with ADHD [31]. In this study, there was indeed a significant positive correlation between the AA:EPA ratio and severity of illness.

Although the EPA and DHA supplementation dosages used in this study were high compared to previous studies with children, there was no serious adverse effect except one case of loose stools that was corrected with a lower dose. Young et al. [32] supplemented adults with ADHD with high-dose EPA/DHA concentrates (approximately 36 g EPA and DHA per day) with no reported serious adverse effects other than loose stools and fishy burps. The average AA:EPA ratio after 12 weeks of the high-dose EPA/DHA supplementation in adults with ADHD was 1.4 ± 0.6 [32]; however, behavior was not assessed in this study.

Stevens et al. [11] supplemented children with ADHD and thirst/skin symptoms with 480 mg DHA, 80 mg EPA, 40 mg AA and 60 mg GLA per day. At these levels, the AA:EPA ratio was reduced to15.19 after four months [11], which remains 2.5 times greater than the mean AA:EPA ratio obtained in this study. Stevens et al. [11] did monitor behavior and found improvements in conduct assessed by the parents and attention assessed by the teachers in the PUFA group compared to olive oil placebo. When assessed clinically, the parental rating scales were also evaluated based on diagnostic criteria, and a significant PUFA treatment effect was reported for oppositional/defiant disorder. The findings by Stevens et al. [11] supports our data in that we also found improvements in oppositional behaviors rated by the parents and improvements in both oppositional/defiant behaviors and conduct assessed clinically by the psychiatrist, however, a psychiatrist did not assess behavior in Stevens et al.'s study.

This study found a statistically significant improvement in the psychiatrist's report of inattention, hyperactivity, oppositional/defiant behavior and conduct disorder based on the ADHD SC-4 questionnaire. Scores for inattention, hyperactivity and oppositional/defiant behavior continued to improve from week four to eight, even with the EPA/DHA concentrate dosage adjustment. The dosage adjustment, however, did not bring the AA:EPA ratio above 3 suggesting the importance of monitoring fatty acids and the AA:EPA ratio in particular rather than EPA/DHA dosage alone. The severity of illness scale demonstrated a positive improvement from an average of moderately symptomatic to mildly symptomatic. This improvement was similar regardless of medication use or lack there of. The percent change in severity of illness also correlated with percent decrease in the AA:EPA ratio, suggesting a connection between the clinical improvement observed by the psychiatrist and the improvements in the AA:EPA ratio.

Data from Stevens et al. [11] in children and Young et al. [32] in adults with ADHD suggest that greater amounts of both EPA and DHA may be required to decrease the AA:EPA ratio to between 1.5 and 3. The mean AA:EPA ratio at the end of this study was 5.95 ± 7.35 for all participants. When the two participants who were non-compliant were removed, the AA:EPA ratio was 2.52 ± 2.91, suggesting a daily dose between 8.1 g and 16.2 g of EPA/DHA concentrate may be appropriate to decrease the AA:EPA ratio to between 1.5 and 3 and to observe improvements in behavior in children with ADHD.

There are a number of limitations to this pilot study and therefore interpretation of results requires caution. The study is limited in that there was no placebo group for reference comparisons as this was a pilot study to determine appropriate dosage for protocol adherence and to maintain AA:EPA levels between 1.5 and 3. Dietary intake was not recorded at baseline or monitored throughout the study; therefore, we are unable to decipher intake of fatty acids from the diet. Also related to diet, we advised the children to eat more fruits and vegetables and consume meals and snacks that are balanced with protein, carbohydrates (preferably fruit and vegetables) and "healthy" monounsaturated fats. Advice for following both a "healthy diet" and high-dose fish oil supplementation may have been confounding factors. However, the dose-response relationship between percent change in AA:EPA ratio and the reduction in the severity of ADHD suggest the behavioral changes were due to, at least in part, the intake of high-dose EPA/DHA concentrates. The lack of behavioral change or regression to pre-study status in those subjects who were least compliant to supplementation also suggest that behavioral changes were associated with intake of the LC omega-3 fatty acids.

EPA/DHA concentrate dosage adjustments themselves can be viewed as a limitation since some, but not all participants' daily intake dosage was modified at week four. The supplement intervention adjustment was based on the AA:EPA ratio, therefore those whose AA:EPA ratio dropped below the goal range was adjusted upward and by using this ratio as our goal, we also avoided most adverse events. The lack of a proper means to monitor supplement intake, such as weight of returned bottles, was also a limitation of this study. However, this was compensated for by use of isolated plasma phospholipids levels as a means to monitor protocol adherence.

## Conclusion

Although this was a small one-arm study, the results are encouraging as they suggest that high-dose EPA and DHA (up to 16.2 g per day) can be given to children with good adherence. Also, our results concur with trends and significant findings of some, but not all studies of PUFA supplementation in children with ADHD or related symptoms [8,10,11]. The inconsistent findings from previous studies and our results suggest that greater dosages of EPA are needed to decrease the AA:EPA ratio to levels similar to the Japanese population and to observe significant behavioral improvements. The findings of this study suggest that children with ADHD and a high AA:EPA ratio might be responsive to treatment with EPA and DHA supplementation to bring the AA:EPA ratio to below 3.

The preliminary results found in this pilot study warrant future randomized, placebo-controlled, double blind studies that use participant's AA:EPA ratios to determine EPA/DHA supplementation dosage for adjunct treatment of ADHD in children.

## Competing interests

BS is a stockholder and president of Zone Labs Inc; HLH is a stockholder and employee of Zone Labs Inc.

## Authors' contributions

PJS was involved with the design of the study and carried out all psychological testing. EMH was involved with the design of the study. HLH performed the statistical analysis and drafted the manuscript. BS conceived the study and helped to draft the manuscript. All authors read and approved the final manuscript.
